# Toxigenic Properties of *Yersinia enterocolitica* Biotype 1A

**DOI:** 10.3390/toxins14020118

**Published:** 2022-02-05

**Authors:** Aleksandra Platt-Samoraj

**Affiliations:** Department of Epizootiology, Faculty of Veterinary Medicine, University of Warmia and Mazury, 10-718 Olsztyn, Poland; platt@uwm.edu.pl

**Keywords:** *Yersinia enterocolitica*, biotype 1A, toxins, YST-b, YSY-c, insecticidal toxins

## Abstract

*Yersinia (Y.) enterocolitica*, an etiological agent of yersiniosis, is a bacterium whose pathogenicity is determined, among other things, by its ability to produce toxins. The aim of this article was to present the most important toxins that are produced by biotype 1A strains of *Y. enterocolitica*, and to discuss their role in the pathogenesis of yersiniosis. *Y. enterocolitica* biotype 1A strains are able to synthesize variants of thermostable YST enterotoxin and play a key role in the pathogenesis of yersiniosis. Biotype 1A strains of *Y. enterocolitica* also produce *Y. enterocolitica* pore-forming toxins, YaxA and YaxB. These toxins form pores in the cell membrane of host target cells and cause osmotic lysis, which is of particular importance in systemic infections. Insecticidal toxin complex genes have been detected in some clinical biotype 1A strains of *Y. enterocolitica*. However, their role has not yet been fully elucidated. Strains belonging to biotype 1A have long been considered non-pathogenic. This view is beginning to change due to the emerging knowledge about the toxigenic potential of these bacteria and their ability to overcome the defense barriers of the host organism.

## 1. Introduction

**Figure 1:**
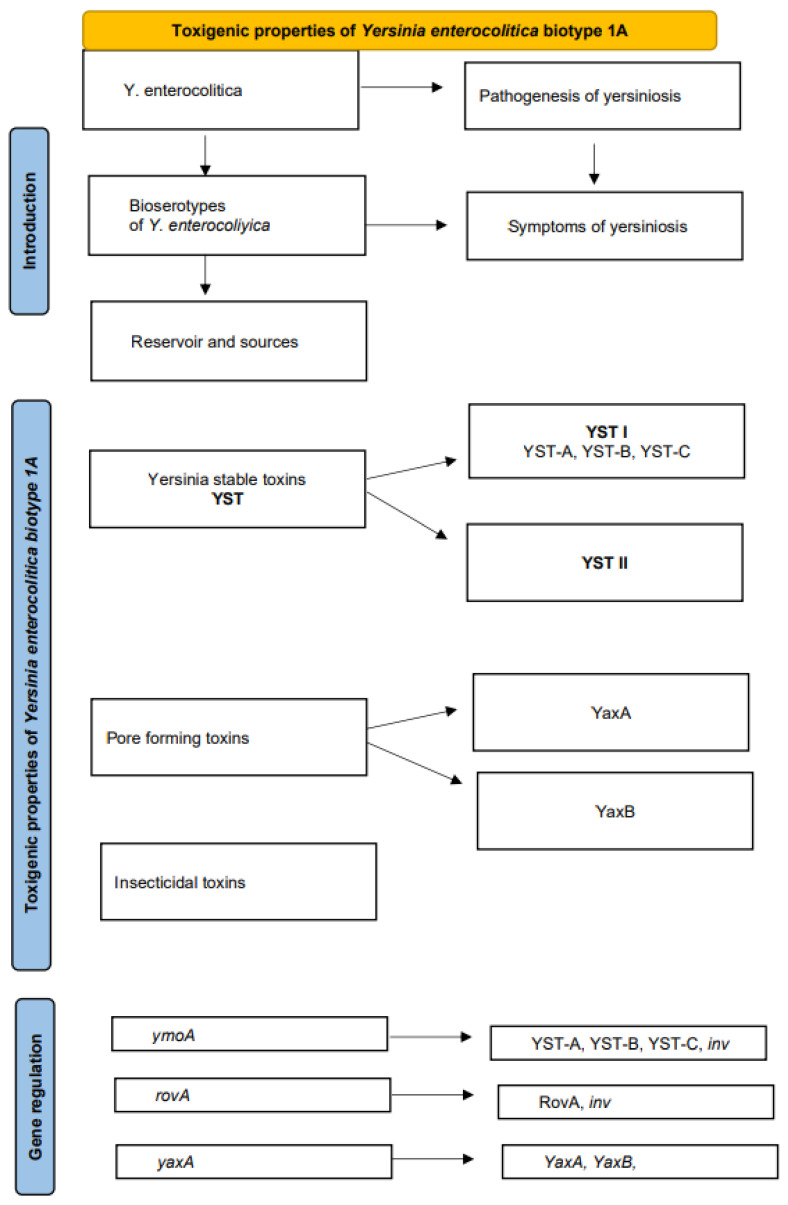
Scheme of the content of the review.

Bacterial toxins are molecules produced by a wide variety of bacteria that attack host cells. Microorganisms can produce different types of toxins. The production of enterotoxins is an important virulence characteristic in most enteropathogens. Enterotoxins play a key role in host–pathogen interactions and are often responsible for the most severe symptoms of disease. Bacterial toxins have various functions in the pathogenesis of food poisoning. In some bacteria, they are mainly a weapon against phagocytes [1]. Macrophages and neutrophils are significant mediators of innate immunity in early stages of bacterial infection, and they are able to remove the pathogen through phagocytosis and subsequent digestion [2]. Bacterial toxins are capable of disrupting enzymatic processes in the attacked host cells [3]. Bacteria also produce toxins that enable them to survive in arthropods, which act as vectors. The function of some bacterial toxins, many of which are detected by accident, has not been elucidated to date [1]. *Yersinia* (*Y.*) *enterocolitica* belongs to a group of microorganisms where the ability to produce toxins is a crucial determinant of pathogenicity. This review article focuses on the significance of toxins produced by this pathogen, their functions, mechanisms of production and impact on the course of yersiniosis (Figure 1).

*Y.enterocolitica* is a foodborne zoonotic pathogen that is widespread in the environment and has a complicated epidemiology that has not yet been fully understood. The pathogen is transmitted by the ingestion of contaminated food. Pigs are considered to be the main reservoir of pathogenic strains for humans, but many species of farm, free-living, and companion animals are susceptible to infection [4]. In most affected animals, the infection is asymptomatic, and the pathogen is abundantly excreted with feces and contaminates the environment. *Y. enterocolitica* is highly resistant to adverse conditions, and it can easily adapt to the environment outside the host organism. The pathogen is able to survive within a pH range of 4.2 to 9 [5] and in water with up to 7% salinity [6,7,8]. Above all, *Y. enterocolitica* has unusual psychotropic properties. It can survive within a wide range of temperatures, especially at low temperatures, and it can compete with most foodborne pathogens that have a preference for higher temperatures. *Y. enterocolitica* is able to grow at 28–29 °C and can survive freezing conditions [9,10]. Temperature is an important signal in the regulation of virulence in *Yersinia* spp. Some toxins are produced mainly at 37 °C and are suppressed at temperatures lower than body temperature. An inverse relationship can be observed in the case of insecticidal toxins that are produced by certain strains of *Y. enterocolitica*. It has been shown that production of these toxins takes place at temperatures below 15 °C, decreases significantly at 25 °C, and disappears at 37 °C [3,11,12].

*Y. enterocolitica* is the agent of yersiniosis, a disease that is most often referred to as acute gastroenteritis. However, yersiniosis can have diverse symptoms that are not limited to the gastrointestinal tract. The course of yersiniosis is determined mainly by the age of the infected host. In adults, most infections are asymptomatic or mild. Yersiniosis accompanied by gastroenteritis is typically encountered in children up to 5 years of age [13]. In animals, symptomatic yersiniosis is also most often observed in very young or immunocompromised individuals. The predominant symptoms in humans are fever, abdominal pain, and diarrhea. In the elderly, in addition to symptoms resembling appendicitis, severe parenteral forms may appear, often of a chronic nature, such as erythema nodosum, or micro abscesses in internal organs [14]. Severe sepsis may occur in generalized infections [15]. *Y. enterocolitica* has also been found to be implicated in autoimmune diseases such as microscopic colitis, reactive arthritis, and Crohn’s disease [16].

Not all strains of *Y. enterocolitica* attack the host organism with equal force. More than 60 serotypes of *Y. enterocolitica* belonging to six biotypes—1A, 1B, 2-5—have been identified based on the differences in the structure of the somatic O—antigen. Pathogenicity is associated belonging to the bioserotype. In clinical practice, bioserotype 1B/O:8 is considered most pathogenic, and bioserotypes 2/O:5.27, 2/O:9, 3/O:3 and 4/O:3 are most commonly isolated (Table 1) [17]. Strains belonging to biotype 1A are defined as non-pathogenic. They are commonly isolated from the environment and from healthy animals and humans. However, recent studies have demonstrated that some biotype 1A strains can cause symptoms no different from those caused by pathogenic strains [18,19,20].

Pathogenic strains of *Y. enterocolitica* are very often isolated from pork. Microbial contamination of carcasses usually occurs in slaughterhouses, despite the introduction of strict sanitary protocols during slaughter to reduce the risk of meat contamination [23,28].

Secondary contamination of refrigerated food with *Y. enterocolitica* also poses a significant risk. The above is facilitated by the growing demand for vacuum-packaged products that are intended for prolonged storage in refrigerators [9,27]. The consumption of raw or undercooked pork is yet another important risk factor [28,29].

## 2. Virulence of *Y. enterocolitica*

The pathogenicity of *Y. enterocolitica* is associated with the presence of the highly conserved 70 kb virulence plasmid pYV and certain chromosomally encoded proteins [30]. For many years, the presence of pYV has been considered evidence of the pathogenicity of *Y. enterocolitica*. This plasmid determines the production of virulence factors, including YadA (Yersinia adhesin A), adhesive proteins facilitating the penetration of the host cell, or the Ysc-Yop type III secretion system (TTSS), including the YopE protein that is cytotoxic to eukaryotes, exhibits antiphagocytic activity and conditions resistance to complement-mediated killing [31]. pYV plasmids confer resistance to phagocytosis and lysis, which enables bacteria that carry these plasmids to proliferate outside cells in host tissues. However, plasmids are unstable structures, and strains belonging to biotype 1A generally do not harbor plasmids. Some strains that do not carry plasmids or have lost them are still able to penetrate host cells. The above indicates that plasmids are not the only mechanism responsible for encoding factors that condition cell penetration in a process known as internalization [32,33,34].

Chromosomal virulence genes are very important elements that determine the pathogenic abilities of *Y. enterocolitica.* These include the attachment invasion locus (*ail*) gene that encodes outer membrane proteins responsible for adhesion, the invasin (*inv*) gene, and the *yst* gene that encodes Yersinia thermostable enterotoxins [35]. Particularly virulent strains of biotype 1B also harbor high-pathogenicity islands (HPI) associated with the iron acquisition system [36,37]. However, the ability to multiply rapidly in the host organism and the production of thermostable toxins play the most important role in the pathogenesis of *Y. enterocolitica* infections.

## 3. Yersinia Stable Toxin—YST

pYV plasmids undoubtedly play an important role in the pathogenicity of *Y. enterocolitica*, but recent research has shown that Yersinia stable toxin (YST enterotoxin) is an equally important virulent factor. This enterotoxin is soluble in methanol, and it can survive boiling for 10 min [38]. In bacterial cells, the production of YST enterotoxin is controlled by *yst* chromosomal genes [39]. It is believed that YST enterotoxin plays a key role in the etiology of diarrhea. Bacterial toxins are a difficult object of research. In the case of YST enterotoxins, the suckling mouse assay (SMA) was initially used to study the expression of *yst* genes, but this test has stirred ethical controversy in recent years. YST enterotoxins are also detected with the use of the ligated rabbit ileal loop test, ELISA, and the Chinese hamster ovary cell culture test [40]. In vivo toxin expression does not always correlate with the actual enteropathogenicity of the test strain. For a long time, research was hampered by the fact that in vitro enterotoxins can be detected after the incubation of bacterial cultures at a neutral pH and temperatures below 30 °C, which is lower than body temperature. In addition, only some strains containing the *yst* gene produce enterotoxins [33,41]. Three types of YST I enterotoxins (A, B and C) encoded by *ystA, ystB* and *ystC* genes, respectively, as well as the poorly understood and biologically active YST II enterotoxin with a completely different mechanism of action have been classified to date. The gene encoding the production of YST II enterotoxin has not yet been identified [18]. The optimal in vitro conditions for the synthesis of YST enterotoxin in *Y. enterocolitica* strains are 26 °C and neutral pH. YST can be also synthesized at 37 °C and pH 7.5, i.e., under conditions that prevail in the final section of the ileum [42]. Therefore, the role of YST I enterotoxin in the pathogenesis of yersiniosis remained questionable for many years. Considerable progress has been made after Singh and Virdi [43] had proposed a novel method for synthesizing enterotoxins in vitro. The cited authors analyzed the properties of *ystB*, and they extended the bacterial culture to 144 h and increased pH to 7.5. As a result, *Y. enterocolitica* biotype 1A isolates began to produce enterotoxins at 37 °C, i.e., under conditions that prevail in the ileum. The above authors also demonstrated that nearly all *Y. enterocolitica* biotype 1A strains isolated from clinical cases of yersiniosis accompanied by diarrhea were capable of producing YST enterotoxin. In contrast, only a small number of non-clinical environmental strains possessed such abilities. This observation confirmed that YST enterotoxin plays a key role in the pathogenesis of yersiniosis.

YST is produced by *Y. enterocolitica* after reaching the final part of the small intestine. Under the influence of high osmolarity, low alkaline pH and temperature 37 °C, the expression of the chromosomal *yst* gene is activated [42]. The (immature) primary form of the toxin is formed, which is a small protein (71 amino acids). It contains 18-amino acid N-terminal signal sequence. At the opposite end of the YST molecule, there is a 30 amino acid C-terminal domain, which is responsible for the biological function of this enterotoxin [44]. The signal sequence is cut off during transport across the cytoplasmic membrane. Upon transition to the periplasm, the C-terminal domain is released from 22 amino acid central sequence. Mature particles (final form) of enterotoxin are secreted into the intestinal lumen [45].

**Figure 2:**
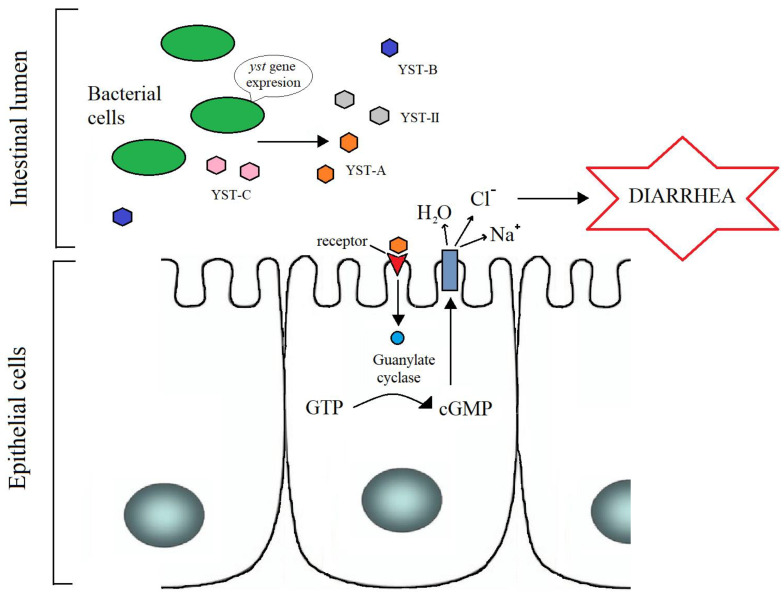
Scheme of action of Yersinia-stable toxins in an infected organism.

Research has shown that YST I enterotoxins produced by *Y. enterocolitica* are biologically and antigenically analogous to STI (Shiga Toxin I) enterotoxins (STa and STb) produced by *E. coli.* STI enterotoxins cause similar changes in cell cultures and rely on the same mechanism of action by stimulating the activation of guanylate cyclase, which increases the concentration of cyclic guanosine monophosphate (cGMP) in epithelial cells and leads to fluid accumulation in the intestine. YST I and STI enterotoxins have the same molecular weight and analogous resistance to temperature and acids [38,41,42,43]. Enterotoxins provoke diarrhea, which is the main cause of mortality in yersiniosis (Figure 2) [13,14,26].

YST enterotoxins produced by *Y. enterocolitica* stimulate the activation of guanylate cyclase, which increases the concentration of cyclic guanosine monophosphate (cGMP) in epithelial cells and leads to fluid accumulation in the intestine. Enterotoxins provoke diarrhea, which is the main symptom of yersiniosis.

### 3.1. YST-A

Classic pathogenic strains of *Y. enterocolitica* contain the *ystA* gene and can, therefore, produce YST enterotoxin [44]. The enterotoxin is synthesized as a polypeptide chain composed of 30 amino acids of the C-terminal domain, which contains a mature component of the toxin and an 18-amino acid N-terminal signal sequence [17]. YST-A is produced by strains of *Y. enterocolitica* belonging to biotypes 1B and 2-5.

### 3.2. YST-B and YST-C

**Figure 3:**
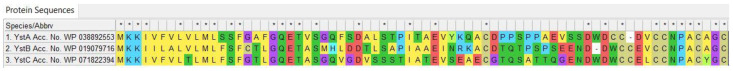
Comparison of amino acid sequences of YST-A, YST-B and YST-C proteins. Common homologous regions for YST-A, YST-B and YST-C are marked with asterisks and solid colors. The amino acids are marked with the following symbols: A—alanine, C—cysteine, D—aspartic acid, E—Glutamic acid, F—Phenylalanine, G—glycine, I—isoleucine, K—lysine, L—leucine, M—methionine, N—asparagine, P—proline, Q—glutamine, R—arginine, S—serine, T—threonine, V—valine, W—tryptophan, Y—tyrosine. Performed with MEGA X: Molecular Evolutionary Genetics Analysis across computing platforms [47].

*Y. enterocolitica* strains belonging to biotype 1A produce mainly enterotoxin YST-B and, much less frequently, YST-C [46]. The DNA sequences of *ystA* and *ystB* genes are characterized by 73.5% homology, and the homology between *ystA* and *ystC* is identical. The homology between YST-A and YST-B amino acid sequences is 57% and between YST-B and YST-C is 60% (Figure 3) [41].

For many years, strains belonging to biotype 1A were considered to be clearly non-pathogenic. However, the pathogenicity of biotype 1A was questioned when Grant et al. [33,48] demonstrated that some biotype 1A strains of *Y. enterocolitica* attacked intestinal epithelial cells and were not killed by macrophages. These observations were attributed to the production of variants of YST I toxin, mainly YST-B and, far less often, YST-C. However, the role of these enterotoxin variants in the pathogenicity of *Y. enterocolitica* is still poorly understood, and their importance in the pathogenesis of yersiniosis has been questioned for a long time. Approximately 85% of biotype 1A strains of *Y. enterocolitica* harbor the *ystB* gene, which encodes the synthesis of YST-B. However, the above does not imply that all biotype 1A strains produce YST.

**Figure 4:**
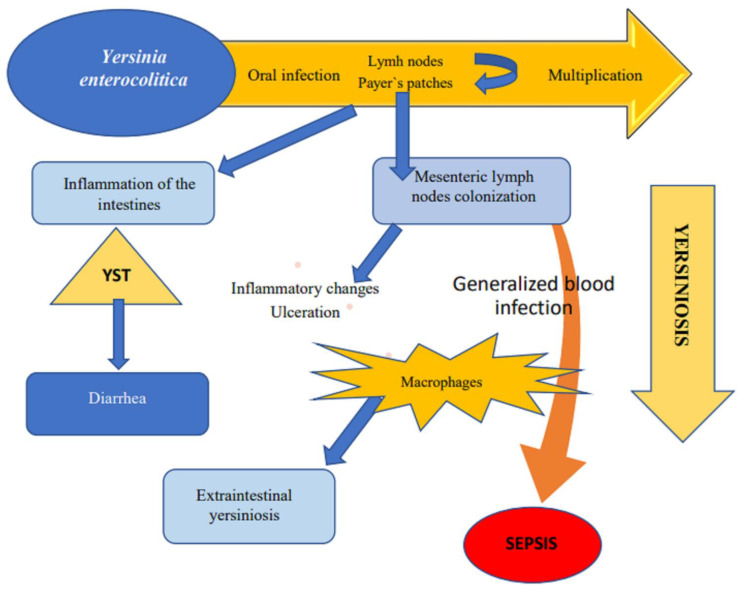
Pathogenesis of yersiniosis.

The ability of biotype 1A strains of *Y. enterocolitica* to cause disease provides indirect evidence that YST enterotoxin plays an important role in yersiniosis. This observation suggests that the disease can be caused by strains that had previously been considered non-pathogenic, do not harbor the pYV plasmid or chromosomal virulence genes such as *ail*, *ystA*, *inv* and HPI, or carry *ystA* only sporadically. Biotype 1A of *Y. enterocolitica* strains isolated from clinical cases of gastritis and enteritis were able to colonize the host’s gastrointestinal tract, both the small and the large intestine, and replicate in enterocytes [33]. Some *Y. enterocolitica* biotype 1A strains were resistant to macrophage killing [20,48]. Nearly 90% of clinical biotype 1A strains produced positive results in the suckling mouse assay, which points to their toxigenic potential (Figure 4) [41].

*Yersinia enterocolitica*, after entering the host organism, adhere to the surface of the intestine. YST enterotoxins induce fluid accumulation in the intestinal lumen, leading to diarrhea. In most cases, the infection is limited to the intestines, but during the process of endocytosis, pathogens penetrate the intestinal wall, and they are transported across mucous membranes to Payer’s patches and to mesenteric lymph nodes with lymphatic fluid. Lymph nodes become inflamed, which can lead to generalized infection of the blood.

The incidence of clinical cases of yersiniosis caused by biotype 1A strains has increased in recent years [8,14,20,49,50]. The *ail* gene, an important virulence marker, has recently been sporadically found in *Y. enterocolitica* biotype 1A strains, but there are doubts that the *ail* itself is a sufficient factor of virulence, and its mechanism of action in *Y. enterocolitica* biotype 1A strains has not yet been established [24]. Therefore, it can be assumed that the pathogenicity of *Y. enterocolitica* biotype 1A results from the expression of the *ystB* gene conditioning the production of YST-B enterotoxin. There is evidence to indicate that *ystB* is the main factor responsible for diarrhea in infections caused by biotype 1A *Y. enterocolitica* strains [43]. Some of the symptoms elicited by these strains are identical to those caused by classic pathogenic strains. Biotype 1A *Y. enterocolitica* strains have been found to cause nosocomial or foodborne outbreaks, as well as parenteral forms of yersiniosis [4,50,51,52]. It can be speculated that either current diagnostic methods are more accurate, or the number of *Y. enterocolitica* 1A strains capable of producing enterotoxins is increasing.

YST-B, produced by biotype *Y. enterocolitica* 1A strains, exerts stronger effects than YST-A. The minimum effective dose of purified YST-A toxin is 7.6 pM, while the corresponding dose for YST-B is 0.4 pM. This is a much smaller quantity than that produced by classic pathogenic strains of *Y. enterocolitica,* but it is as potent as STI enterotoxin produced by *E. coli*, where the minimum dose is also 0.4 pmol [41]. The above indicates that YST-B enterotoxin plays a very important role in the pathogenesis of foodborne infections.

In contrast, the *ystC* gene responsible for YST-C, the third variant of YST enterotoxin, has rarely been identified, and mainly in *Y. enterocolitica* biotype 1A strains. YST-C contains 53 amino acids, has a higher molecular weight than YST-A and YST-B, and contains C- and N-terminal chains with 50% similarity to YST at the amino acid level [40]. The existing knowledge about this toxin is still modest.

## 4. Insecticidal Toxins of Biotype 1A *Y. enterocolitica* Strains

Biotype 1A strains of *Y. enterocolitica* form a heterogeneous group of diverse serotypes, many of which do not react with the available diagnostic sera. Tennant et al. [53] examined differences between biotype 1A *Y. enterocolitica* strains from clinical cases and strains isolated from the environment. The compared isolates were divided into two groups based on their DNA sequences [53]. In comparison with non-clinical strains, the potentially pathogenic *Y. enterocolitica* biotype 1A strains were characterized by greater ability to invade HEp-2 cell cultures and Chinese hamster ovary cells (CHO), survive in the presence of macrophages and in the gastrointestinal tract of orally infected mice for longer periods of time [48]. The factors responsible for these differences are unknown. Some clinical strains harbored genes corresponding to the insecticidal toxin complex (TC) that is also found in other bacterial species. Toxin complex genes were not identified in non-clinical strains [54]. *Yersinia pestis*, the causative agent of plague, is a bacterial species that carries TC gene homologues. It is generally believed that TC genes enable this bacterium to survive in the intestines of fleas which are the vector of plague [3]. The role played by TC in biotype 1A strains of *Y. enterocolitica* remains unknown. Deng et al. [55] suggested that these genes may contribute to the virulence properties of some *Y. enterocolitica* biotype 1A strains.

In *Photorhabdus luminescens,* a bacterial insect pathogen of the *Enterobacteriaceae* family, TC genes encode high-molecular-weight toxins that are capable of killing insects [56]. However, TC genes have also been identified in bacteria that are not related to insects. In addition to *Y. enterocolitica* biotype 1A strains, TC genes have been detected in *Y. pseudotuberculosis*, selected plant bacteria and bacteria that are part of the oral microflora [53]. It is possible that in these bacteria, TC genes do not encode insecticidal toxins, but serve other as yet unknown purposes. Toxin complex proteins may affect the enterotoxic activity of some biotype 1A strains of *Y. enterocolitica* [53].

## 5. Pore-Forming Toxins YaxA and YaxB

Pathogenic strains of *Y. enterocolitica* can also produce YaxA and YaxB cytotoxins, also known as *Y. enterocolitica* pore-forming toxins [57]. These toxins are regulated by the RovA gene [58]. YaxA and YaxB toxins form pores in the membrane of host target cells and cause osmotic lysis, which plays a very important role in systemic infections. In experiments performed on the murine model, the most severe damage was observed in the spleen [58]. Interestingly, full lytic activity occurs only when YaxA and YaxB work together. Toxins with analogous effects are also found in bacteria of the genera *Bacillus* and *Xenirhabdus* [59].

The mechanisms underlying the production and functions of YaxA and YaxB remain insufficiently investigated. These toxins do not have a defined signal sequence. They could be released spontaneously as a result of bacterial lysis, or they could be released from bacteria through outer membrane vesicles. YaxA and YaxB can bind nonspecifically to host cells through cholesterol, which is a universal component of cell membranes [57]. The presence of cytotoxins YaxA and YaxB may be caused by the death of bacterial cells at the site of infection. This may lead to changes in the cytokine expression, thus influencing the innate immune response of the host, which may facilitate the survival of the remaining bacteria [57].

## 6. Gene Regulation

*Y. enterocolitica* cells may contain different genes that control the production of various toxins. However, the above does not imply that all toxins are produced by a given strain of bacteria. The synthesis of toxins depends mainly on the expression of genes responsible for the production of proteins. These processes, in turn, are associated with complex systems of genetic regulation that are usually conditioned by regulatory genes [60]. The expression of a gene encoding the production of one toxin is often determined by several regulatory genes that control the synthesis of histone-like proteins, which, in turn, may be responsible for the expression of more than one gene [59,61]. These processes are complex, and they have not yet been fully elucidated.

Research has demonstrated that not all biotype 1A strains harboring *ystB* produce YST-B toxin due to the presence of “silent genes”. According to recent studies, Yersinia-modulating protein YmoA encoded by the *ymoA* gene is the main regulator of *yst* gene expression [35]. A certain analogy can be drawn between *ymoA* gene expression and the production of YST-B. *YmoA* gene activity was more common in strains isolated from clinical cases and from pigs than in environmental strains [33,43]. YmoA is a nucleoid-associated protein with 88% homology to the regulator of high hemolysin activity (Hha), a histone-like protein that occurs in *E. coli* and *Salmonella.* These proteins play an important role as structural proteins and regulators of gene expression [61]. The *ymoA gene* is found in the chromosome of all *Y. enterocolitica* strains, and it was also detected in strains belonging to biotype 1A [40]. YmoA is one of the main modulators of gene expression in response to environmental conditions, mainly temperature, and it is involved in the negative transcription of virulence markers [62]. The *ymoA* mutation unlocks the silencing of the *yst* gene and stimulates the production of enterotoxins [60]. In some *Y. enterocolitica* strains that carry the *yst* gene, the absence of enterotoxic properties could be attributed to the inhibitory effect of the *ymoA* gene on *yst* expression [63]. YmoA also modulates the expression of *inv* genes, virulence factors that are responsible for the invasive abilities of *Y. enterocolitica* and *Y. pseudotuberculosis* [57].

RovA is yet another histone-like protein that is encoded by the *rovA* gene. RovA has numerous functions, including the regulation of the *inv* gene. YmoA and H-NS, which form the so-called repressive complex, exert the same effect. RovA and H-NS/YmoA form a global regulatory system used by *Y. enterocolitica*. Additionally, *yaxA* and *yaxB* genes encoding the production of YaxA and YaxB toxins are regulated by the same system [57].

## 7. Source of Food Poisoning during Refrigerated Storage

*Y. enterocolitica* has been isolated from various foods, mainly products stored in a refrigerator. *Y. enterocolitica* is a pathogen of emerging concern [4,7] due to its ability to grow at low temperatures. The production of toxins responsible for the most severe symptoms is triggered by low temperature, followed by thawing [7].

*Y. enterocolitica* has also been isolated from products that undergo pasteurization, mostly milk and cottage cheese [64]. The above implies that some bacterial strains can survive the pasteurization process, although secondary contamination cannot be excluded [29]. On the other hand, there are no reports of heat-resistant strains of *Y. enterocolitica*. This observation gives serious cause for concern, because pasteurization kills most bacteria, and in refrigerated products that are contaminated with *Y. enterocolitica,* the pathogen is able to grow at a temperature of 4 °C without competition. *Y. enterocolitica* has also been identified in vacuum-packaged products with a pH of 6, including lettuce, ready-to-eat vegetables and juices stored at around 3 °C [9,27,65,66]. In vacuum packaging, *Y. enterocolitica* grew at all storage temperatures at a rate similar to or faster than the microflora that causes food spoilage [27]. In foods with a neutral pH that are stored in the refrigerator at a temperature of around 5 °C, bacterial counts can increase from 10/mL to 2.8 × 10^7^/mL within 5 days. The production of toxins is influenced by the temperature of bacterial growth. A toxin-producing *Y. enterocolitica* strain synthesized thermostable enterotoxin in milk at a temperature of 25 °C, but not 4 °C [64].

## 8. Effect of Toxins on the Pathogenesis of Yersiniosis

Due to its resistance to low pH, *Y. enterocolitica* is able to overcome the barrier of the acidic environment in the stomach and then can be transferred to the intestine [6]. In the pathogenesis of yersiniosis, not only enterotoxins, but also other virulence factors, interact with each other to enable the pathogen to survive in the host organism.

After entering the host organism, bacteria adhere to the surface of the intestine through the YadA protein. Adhesin binds to beta 1 integrin of the mucous membrane of the terminal ileum [67]. Some biotype 1A strains of *Y. enterocolitica* are able to invade intestinal epithelium cells even in the absence of YadA protein [8,18,34]. YST-B is a major contributor to enterotoxigenicity, and the direct cause of fluid accumulation in the intestinal lumen [43]. During the process of endocytosis, bacteria penetrate the intestinal wall and merge with M cells. Pathogens that come into contact with M cells are transported across mucous membranes to Payer’s patches and to mesenteric lymph nodes with lymphatic fluid. Lymph nodes become inflamed, which can lead to generalized infection of the blood [52]. In such cases, mortality reaches 54.5% [16].

Toxins not only affect enterocytes and induce diarrhea, but also influence macrophage functions. In response to infection, macrophages secrete chemokines which attract monocytes and neutrophils to the site of infection. Monocytes and neutrophils release pro-inflammatory cytokines as well as additional chemokines that enhance the action of macrophages. The progression of infection is largely dependent on phagocyte responses. Toxin-producing pathogens that compromise phagocyte functions are not eliminated, and the disease can develop [68].

Some pathogens attack macrophages and neutrophils mainly by secreting toxins that either cause irreversible damage leading to the death of phagocytes or strongly disrupt intracellular signaling pathways, block phagocytosis or modulate inflammatory processes, for example, by controlling the expression of chemokines and cytokines [69].

Yersinia stable toxin not only enables *Y. enterocolitica* to survive, but also to multiply in macrophages and use them as a means of transport to various organs distant from the place of infection in the host body [17,30]. The mechanisms by which *Y. enterocolitica* evades killing by macrophages are still poorly understood [70,71].

## 9. Conclusions

The role of toxins, especially YST enterotoxins, as virulence factors in the pathogenesis of yersiniosis is underestimated. YST enterotoxins are crucial virulence factors in *Y. enterocolitica*, and the most important virulence factors in biotype 1A strains. The differences in the pathogenicity of biotype 1A strains result from regulatory genes that inhibit the expression of *yst* genes in some strains. Further research is needed to elucidate the effect of other toxins produced by *Y. enterocolitica* biotype 1A strains on the pathogenesis of yersiniosis.

Most procedures for diagnosing the causes of food poisoning focus on classic pathogenic strains of *Y. enterocolitica.* The growing number of reports on clinical cases of yersiniosis caused by *Y. enterocolitica* biotype 1A strains and the emerging knowledge about their pathogenic potential suggest that the incidence of yersiniosis will probably increase in the future. Therefore, biotype 1A strains *Y. enterocolitica* should be considered in the differential diagnosis of intestinal diseases.

## Figures and Tables

**Table 1:** Relationship between bioserotype of *Yersinia enterocolitica* and pathogenic properties and source of isolation.

Biotype	Serotype	Pathogenic Properties	Sources of Isolation
**1A**	O:4, O:5, O:6,31, O:7,13, O:7.8, O:10, O:14, O:16, O:21, O:22, O:25, O:37, O:41, O:46, O:46, O:57, NT	Non-virulent (or conditionally virulent)	Soil [21], surface water, environment, game animals [22], vegetables [23], pigs [24], wild rodents [25], food producing animals [21]
**1B**	O:4.32, O:8, O:13a, O:13b, O:16, O:18, O:20, O:21, O:25, O:41.42, NT	Highly virulent	pigs [23], contaminated vegetables [21], human faeces, rodents [26], pork, milk products [21], refrigerated products [10]
**2**	O:5.27, O:9, O:27	Weakly virulent	Pork products [23,27], milk products [21], asymptomatic pigs‘ tongues [23]
**3**	O:1.2.3, O:3, O:5.27		Pork products, undercooked meat [23], contaminated vegetables [4], pigs [23], refrigerated products [10]
**4**	O:3		Pork products [19], human faeces, pigs [23], pets [4], milk products [21]
**5**	O:3.2.3		Milk products [21], small ruminants, sheep, hares [4], pigs [23]

NT—not typable.

## Data Availability

No new data were created or analyzed in this study. Data sharing is not applicable to this manuscript.
